# PREMATURE MORTALITY IN LGBT VERSUS NON-LGBT VETERANS: THE ROLE OF HEALTH RISK FACTORS AND SOCIAL DETERMINANTS OF HEALTH

**DOI:** 10.1093/geroni/igad104.1647

**Published:** 2023-12-21

**Authors:** Sherry Beaudreau, Julie Lutz, Marcela Otero, Allison Warren, Joseph Goulet

**Affiliations:** VA Palo Alto/ Stanford University School of Medicine, Palo Alto, California, United States; VA Palo Alto Health Care System, Palo Alto, California, United States; Max-Plank Institute for Social Law and Social Policy, Munich, Bayern, Germany; Yale School of Medicine, New Haven, Connecticut, United States; Yale School of Medicine, New Haven, Connecticut, United States

## Abstract

Lesbian, Gay, Bisexual, and Transgender (LGBT) adults experience substantially higher rates of mental health conditions and other health risk factors than non-LGBT adults, but there is limited reseach on how these conditions increase risk for premature mortality. The current study examined this issue among middle-aged and older Veterans ages 40-99 who used Veterans Administration healthcare services from 01OCT2009 to 30SEP2019 (N = 845,122). Earlier age of death by suicide, overdose, or all-causes was hypothesized for LGBT (n = 675,639 ) vs. non-LGBT (n = 675,639) Veterans, and health factors and social determinants of health were predicted as key risk factors of earlier age of mortality. As hypothesized, LGBT Veterans had significantly higher rates of death by suicide, overdose, and all-causes among younger age groups than non-LGBT Veterans. Adjusted risk ratios indicate a significant contribution of mental health conditions (e.g., anxiety/depression), medical comorbidity, pain, smoking history, and military sexual trauma accounting for differential mortality rates by age in LGBT and non-LGBT participants. Social determinants of health, such racial/ethnic minority status and housing instability, were also significant contributors. Findings suggest the need for Whole Health interventions for LGBT Veterans to manage multiple health risk factors contributing to increased mortality risk. Suicide prevention is critical for the many middle-aged and older adults who die from suicide each year, and for LGBT Veterans especially, beginning targeted suicide prevention efforts earlier in middle age may prevent suicide deaths in later life. Lastly, findings implicate subgroups of Veterans who may particularly benefit from outreach and services.

